# The Role of [^18^F]FES PET/CT in Breast Cancer Management: An Umbrella Review

**DOI:** 10.3390/cancers17101644

**Published:** 2025-05-13

**Authors:** Marco Cuzzocrea, Rosa Di Micco, Giorgia Elisabeth Colombo, Stefania Maria Rita Rizzo, Gaetano Paone, Virginia Casati, Turki Alkhaldii, Fatemah Khajah, Claudia Rauh, Maggie Banys-Paluchowsky, Nina Ditsch, Thorsten Kuehn, Oreste D. Gentilini, Giorgio Treglia, Maria Luisa Gasparri

**Affiliations:** 1 Division of Nuclear Medicine, Istituto di Imaging della Svizzera Italiana (IIMSI), Ente Ospedaliero Cantonale (EOC), Via Athos Gallino 12, 6500 Bellinzona and Via Tesserete 46, 6900 Lugano, Switzerland; marco.cuzzocrea@eoc.ch (M.C.); gaetano.paone@eoc.ch (G.P.); giorgio.treglia@eoc.ch (G.T.); 2 Breast Surgery Unit, IRCSS Ospedale San Raffaele, Via Olgettina 60, 20132 Milan, Italy; dimicco.rosa@hsr.it (R.D.M.); turkialkhaldii@gmail.com (T.A.); fmkhajah@gmail.com (F.K.); gentilini.oreste@hsr.it (O.D.G.); 3 Department of Obstetrics and Gynaecology, Chelsea and Westminster NHS Foundation Trust, 369 Fulham Rd., London SW10 9NH, UK; giorgiaecolombo@gmail.com; 4 Department of Gynaecology and Obstetrics, Ente Ospedaliero Cantonale (EOC), Ospedale Regionale di Lugano, Via Tesserete 46, 6900 Lugano, Switzerland; virginia.casati@eoc.ch; 5 Division of Radiology, Istituto di Imaging della Svizzera Italiana (IIMSI), Ente Ospedaliero Cantonale (EOC), Via Tesserete 46, 6900 Lugano, Switzerland; stefaniamariarita.rizzo@eoc.ch; 6 Faculty of Biomedical Sciences, Università della Svizzera Italiana (USI), Via Buffi 13, 6900 Lugano, Switzerland; 7 Department of Gynecology, University Hospital Inselspital Bern, Theodor-Kocher-Haus Friedbühlstrasse 19, 3010 Bern, Switzerland; claudia.rauh@insel.ch; 8 Department of Gynaecology and Obstetrics, University Hospital Schleswig-Holstein, Campus Luebeck, Ratzeburger Allee 160, 23562 Lübeck, Germany; 9 Klinik fuer Gynäkologie und Geburtshilfe, Universitätsklinikum Augsburg, Brustzentrum, Stenglinstraße 2, 86156 Augsburg, Germany; nina.ditsch@uk-augsburg.de; 10 Department of Surgery, Die Filderklinik, Im Haberschlai 7, 70794 Filderstadt, Germany; t.kuehn@filderklinik.de; 11 Universitaetsfrauenklinik Ulm, Prittwitzstraße 43, 89081 Ulm, Germany; 12 Faculty of Medicine, Università Vita-Salute San Raffaele, Via Olgettina 58, 20132 Milan, Italy; 13 Faculty of Biology and Medicine, University of Lausanne, Rue du Bugnon 21, 1011 Lausanne, Switzerland; 14 Department of Gynecology and Obstetrics, Centro di Senologia della Svizzera Italiana EOC, Ospedale Italiano di Lugano, Via Pietro Capelli 1, 6962 Lugano, Switzerland

**Keywords:** breast cancer, [^18^F]FES PET/CT, estrogen receptor, PET, positron emission tomography

## Abstract

**Background/Objectives:** Breast cancer (BC) is the most commonly diagnosed cancer worldwide. Estrogen receptor (ER) status is a key determinant in the diagnosis and treatment of BC. Although immunohistochemistry (IHC) is the gold standard for ER assessment, it has limitations. This umbrella review aims to evaluate the role of 16α-18F-fluoro-17β-estradiol ([^18^F]FES) PET/CT as a non-invasive imaging tool for assessing ER expression and its implications in BC management. **Methods:** A comprehensive search was conducted in PubMed/MEDLINE and Cochrane Library for systematic reviews and meta-analyses published in the last decade. Studies eligible for inclusion evaluated the diagnostic accuracy and clinical utility of [^18^F]FES PET/CT in BC based on a predefined research question “What is the role of fluoroestradiol ([^18^F]FES) PET/CT in breast cancer?”. Data extraction and quality assessment were performed independently by two reviewers using the AMSTAR-2 tool. **Results:** Eight systematic reviews met the inclusion criteria. [^18^F]FES PET/CT demonstrated high sensitivity (81–94%) and specificity (78–95%) in detecting ER-positive lesions. It provided a real-time, whole-body assessment of ER expression, outperforming IHC in detecting functional ER activity. Additionally, [^18^F]FES PET/CT showed promise in predicting treatment response and guiding therapy decisions, particularly in metastatic settings. **Conclusions:** This review highlights the clinical value of [^18^F]FES PET/CT in BC management, offering a non-invasive alternative for ER assessment with high diagnostic accuracy. Its integration into clinical practice may enhance personalized treatment strategies for BC patients.

## Simple Summary

Breast cancer is the world’s most commonly diagnosed cancer and the leading cause of cancer-related death in women. Estrogen receptor (ER) status in tumors plays a key role in determining the treatment for breast cancer. However, assessing ER status can be challenging due to tumor heterogeneity and the limitations of current methods, including tissue biopsies and immunohistochemistry (IHC). This review focuses on the use of [^18^F]FES PET/CT, an imaging method that provides an evaluation of ER activity across the body, potentially offering a non-invasive diagnosis. The aim of this study is to assess how [^18^F]FES PET/CT can improve breast cancer diagnosis and treatment planning, particularly when biopsies are unfeasible or unsafe. The findings could help optimize therapy choices and lead to better management of breast cancer, particularly in cases of recurrence or metastasis.

## 1. Introduction

Breast cancer (BC) is the world’s most commonly diagnosed cancer, with over 2.3 million new patients every year, and is the primary cause of cancer-related mortality in women [1]. More than 70% of BCs express estrogen receptors (ER), making ER status in a primary breast tumor a crucial determinant in initial evaluation and treatment planning. Similarly, a biopsy is recommended when recurrence or metastasis is suspected to confirm the histopathological diagnosis and reassess ER status, enabling selection of the most suitable therapy [2]. However, discrepancies between primary and secondary lesions are possible, and obtaining tumor tissue from metastatic sites is not always feasible or safe [3].

Currently, ER status is primarily assessed through immunohistochemistry (IHC) on tissue samples. While IHC is considered the gold standard, it has notable limitations: it requires invasive sampling, provides limited information due to tumor heterogeneity, and does not assess functional ER activity. Additionally, despite being ER positive by IHC, some tumors develop resistance to endocrine therapy over time [4,5,6,7,8]. The radiolabeled compound 16α-18F-fluoro-17β-estradiol ([^18^F]FES) is a form of estrogen that is able to bind to functionally active ER, thus allowing non-invasive, whole-body evaluation through positron emission tomography (PET)/computed tomography (CT) imaging. Unlike IHC, [^18^F]FES PET/CT provides a comprehensive, real-time assessment of ER activity across all tumor sites [9,10,11], offering a more reliable predictor of therapeutic response. This imaging technique is also useful when biopsy is not technically feasible or safe [12] and when other imaging modalities yield inconclusive results [13]. In this regard, the FDA approval of [^18^F]FES PET/CT in May 2022 marked a significant milestone in the diagnostics management of ER-positive recurrent or metastatic breast cancer [11]. Furthermore, compared to [^18^F]Fluoro-2-deoxy-2-D-glucose ([^18^F]FDG), the current standard PET/CT tracer in BC, [^18^F]FES may enhance the staging of patients with invasive lobular carcinoma [14] or invasive ductal carcinoma with low [^18^F]FDG avidity [15].

We conducted an umbrella review to assess the role of [^18^F]FES in BC management. This review provides a comprehensive evaluation of its utility in detecting breast cancer lesions and its broader clinical implications, drawing on data of the highest level of evidence from systematic reviews and meta-analyses.

## 2. Materials and Methods

This umbrella review was performed according to published rules [16]. The protocol was not registered on PROSPERO or public databases. Based on a predefined research question (“What is the role of fluoroestradiol ([^18^F]FES) PET/CT in breast cancer?”), a search string was created using a combination of “free-text” keywords and Boolean operators. The complete search string used for the literature search was (A) “breast” AND (B) “FES” OR “fluoroestradiol” AND (C) “systematic review” OR “meta-analysis” OR “evidence-based”. An electronic web-based comprehensive search of the medical literature was carried out using the above-listed search string on two different bibliographic databases (PubMed/MEDLINE and Cochrane Library). The last update of the literature search was on 31 August 2024. No language restrictions were applied; however, only evidence-based articles published in the last 10 years were selected. References of the retrieved records were screened, searching for additional systematic reviews or meta-analyses on the selected topic. Regarding the inclusion criteria, only systematic reviews with or without meta-analyses investigating the role of [^18^F]FES PET/CT in breast cancer were eligible for inclusion in our umbrella review. Two review authors (MC and GT) independently performed the literature search and the selection of eligible records by applying the inclusion criteria mentioned above, and the data extraction and the quality assessment of eligible systematic reviews according to the AMSTAR-2 tool [17]. For each selected systematic review (with or without meta-analysis), information was collected, including authors, year of publication, number of original articles included, patients included, and main findings (with a special focus on diagnostic performance data). The results of this umbrella review are reported narratively.

## 3. Results

The comprehensive web-based literature search using PubMed/Medline and Cochrane Library databases retrieved 16 records, with an additional evidence-based article found via reference screening, for a total of 17 records. Following the screening process according to the predefined inclusion and exclusion criteria, 8 systematic reviews and/or meta-analyses were selected for inclusion in this review [12,18,19,20,21,22,23,24]. The main characteristics of selected systematic reviews and/or meta-analyses are presented in Table 1 and summarized below. The quality assessment of included evidence-based articles according to AMSTAR-2 is illustrated in Table 1.

Regarding technical aspects, the median activity of the radiotracer intravenously administered to the patients was approximately 200 MBq. Although a rapid clearance of [^18^F]FES has been described and imaging starting 20 to 30 min after the injection may provide good visualization, the median uptake interval between radiotracer administration and scan acquisition was about 60 min, as reported by Huang et al. [21]. [^18^F]FES uptake was generally estimated by visual analysis and by utilizing semi-quantitative parameters such as SUVmax and SUVmean [12,18,19,21,22].

Immunohistochemistry (IHC) was used as a standard criterion for confirming ER positivity in about half of the studies [12,19,23]. Different reference standards (including other diagnostic imaging modalities and blood sampling) and clinical follow-up data were used in the remaining studies [21].

When considering diagnostic performance, [^18^F]FES PET or PET/CT showed high pooled sensitivity and specificity in evaluating the expression of ER in BC lesions: pooled sensitivity and specificity ranged from 81% to 94% and from 78% to 95%, respectively [12,18,19,20,22,23,24] (Table 2). Notably, there were differences between the various studies in terms of correlation and agreement with a reference standard test (IHC vs. non-IHC). In addition, qualitative and quantitative cut-offs for both [^18^F]FES PET positivity and ER status were not uniform across studies. The pooled detection rate of ER-expressing lesions in patients with BC using [^18^F]FES PET or PET/CT was evaluated by a single meta-analysis, which showed a detection rate of 80% regardless of whether it was based on patients or lesions (Table 2). Conversely, the detection rate based on patients and lesions was 78% and 82%, respectively [21]. However, statistical heterogeneity was reported among the studies included in the selected evidence-based articles [12,20,21]; furthermore, the presence of publication bias was demonstrated [18] (Table 2).

Several subgroup analyses of diagnostic performance of [^18^F]FES PET were performed in some of the included evidence-based articles, taking into account the presence of statistical heterogeneity in the main meta-analyses. Subgroup analyses included correlation and agreement with a reference standard test (IHC vs. non-IHC), type of lesions (breast vs. metastatic lesions) [12], the PET technique (hybrid PET/CT vs. PET only) [19], timing of the imaging (i.e., PET/CT performed for initial staging or restaging), sample size of the study, the prevalence of ductal or lobular BC, the prevalence of bone and liver metastases [20], and tumor size and [^18^F]FES uptake [22]. Overall, as reported in each individual study, no significant statistical differences in the diagnostic performance of the index test were found among these subgroups.

The uptake threshold of [^18^F]FES to detect ER expression has been assessed in several studies [21] using semi-quantitative PET parameters. The threshold of SUVmax in [^18^F]FES PET to identify positive ER-expressing lesions was between 1.5 and 1.82, with a sensitivity of 0.85 and 0.90 and a specificity of 0.79 [21]. In addition, the rate of ER heterogeneity among patients with metastatic breast cancer was 11.1% to 45% when the threshold of SUVmax in [^18^F]FES PET was 1.5 and 11.8% to 34.3% when the threshold of SUVmax in [^18^F]FES PET was 1.82 [21]. Finally, the threshold of [^18^F]FES SUVmean to identify positive ER-expressing lesions was between 1.1 and 1.5 with sensitivity and specificity of 0.85 to 0.94 and 0.75 to 0.94, respectively [21].

The correlation between ER expression tested by IHC and [^18^F]FES uptake was reported in many studies [12,19,21,23], and in all studies that reported this correlation, a moderate to strong agreement was found [18]. [^18^F]FES PET can be employed as a predictive tool for treatment response in breast cancer patients with different types of therapy (i.e., endocrine therapy ± CDK4/6 inhibitor, neoadjuvant endocrine/chemotherapy). Again, quantitative cut-off values for [^18^F]FES PET positivity (i.e., SUVmax and SUVmean values) and/or other quantitative parameters (i.e., [^18^F]FES/[^18^F]FDG ratio) were not uniform across studies. However, the odds of a better metabolic response, assessed by [^18^F]FDG PET/CT, in the [^18^F]FES-positive lesions were 1.44 times better compared with a [^18^F]FES-negative lesion [21]. Furthermore, using the threshold of SUVmax in [^18^F]FES PET to predict the treatment response revealed that the threshold of SUVmax of 1.5 to 2.0 presented 0.45 to 0.60 positive predictive value and 0.78 to 0.81 negative predictive value [21]. Using the threshold of SUVmean in [^18^F]FES PET to predict treatment response showed that the rate of treatment response was between 35% and 40% when SUVmean was greater than 1.5 but there was a 29% rate of predicted endocrine resistance when the SUVmean was less than 1.0 [21].

[^18^F]FES PET was evaluated in some studies to predict disease prognosis among patients with breast cancer. The median time to progression of positive [^18^F]FES PET (73 weeks) was longer than heterogeneous [^18^F]FES uptake (27 weeks) and negative [^18^F]FES PET (15 weeks) in patients who received first-line endocrine therapy plus CDK4/6 inhibitor treatment [21]. However, not all studies were able to predict PFS and OS by using only semiquantitative parameters in [^18^F]FES PET; the threshold of the [^18^F]FES/[^18^F]FDG ratio was also used for predicting the prognosis [21].

In a few studies, [^18^F]FES PET/CT has been used for monitoring response to AI, tamoxifen, fulvestrant, vorinostat, or Z-endoxifen, demonstrating a significant reduction of [^18^F]FES uptake in responders compared to non-responders [21]. The complementary role of [^18^F]FDG and [^18^F]FES PET can be useful for determining early response to hormonal therapy [18].

When [^18^F]FES PET is compared with [^18^F]FDG PET, both diagnostic procedures are effective without statistically significant differences [22]; however, the better performance may depend on the specific features of breast cancer (i.e., ductal carcinoma vs. lobular carcinoma) and the site of metastatic lesions (i.e., liver vs. bone). Interestingly, the sensitivity of [^18^F]FES PET/CT at the time of restaging was significantly higher than that of the same procedure at the time of staging [20,21]. The detection rate of [^18^F]FES PET/CT was similar to that of [^18^F]FDG PET/CT [21]. Patients with ER-negative breast cancer would be expected to have higher [^18^F]FDG uptake (probably due to an increase in GLUT-1 expression) than patients with ER-positive tumors [18].

Studies showing a higher number of [^18^F]FES-positive lesions often analyzed patients affected by lobular breast cancer or patients with a high prevalence of bone metastases. Studies reporting a higher number of [^18^F]FDG-positive lesions often included patients affected by ductal carcinoma and those with liver metastases [20].

Among patients with breast cancer, the pooled median of SUVmax in [^18^F]FES PET based on the patient and lesion was 4.71 and 3.10, respectively, which were both lower than the [^18^F]FDG SUVmax. In addition, the pooled median SUVmean in [^18^F]FES PET in breast cancer patients was 2.10, which was also lower than that of [^18^F]FDG PET [21].

Interestingly, a change of treatment or management was reported in 20–48% of breast cancer patients performing [^18^F]FES PET/CT [21,22].

Regarding the safety of the test, Chae et al. reported that 10% of patients felt pain with drug injection [24]. However, no side effects have been reported [19]. The effective dose equivalent of [^18^F]FES PET/CT was 0.022 mSv/MBq. The organ that received the highest dose was the liver (0.13 mGy/MBq), followed by the gallbladder (0.10 mGy/MBq) and the urinary bladder (0.05 mGy/MBq]) [21].

## 4. Discussion

To our knowledge, this is the first umbrella review conducted to provide a comprehensive assessment of the clinical value of [^18^F]FES PET/CT in the management of breast cancer. Our findings underscore the growing evidence supporting [^18^F]FES PET/CT as a valuable and safe tool for non-invasive evaluation of ER expression and activity in breast cancer lesions.

The diagnostic performance of [^18^F]FES PET or PET/CT was assessed across all included reviews (Table 2). Our findings consistently demonstrate that [^18^F]FES PET or PET/CT is highly effective in identifying ER-positive lesions, with sensitivity and specificity pooled values ranging from 81 to 94% and from 78 to 95%, respectively. Moreover, the eight included reviews predominantly exhibited moderate to high methodological quality (Table 1). Despite differences in terms of correlation and agreement with a reference standard test, almost all studies evaluated the diagnostic performance of [^18^F]FES, and in all cases a moderate to strong agreement was found [12,19,21,23]. Huang et al. reported an 80% (95% CI, 75–85%) detection rate of malignancy regardless of whether it was based on patients or lesions [21]. As a result, [^18^F]FES PET could potentially replace IHC, offering low rates of false negatives and false positives [18]. However, the detection rate was not reported in other reviews. Similarly, statistical heterogeneity and publication bias were evaluated in a minority of the included studies (Table 2).

Evaluating ER status through functional imaging can enhance the detection of tumor heterogeneity and provide a more accurate prediction of response to endocrine therapy [18]. Furthermore, monitoring ER status during treatment may provide valuable insights into survival outcomes [25]. In detail, higher FES uptake is associated with better response to ER-targeted therapy and potentially improved survival outcomes [26,27]. Our review found that [^18^F]FES-positive lesions were significantly more likely to show a better metabolic response on [^18^F]FDG PET/CT compared to [^18^F]FES-negative lesions [21]. Our findings confirm that [^18^F]FES PET serves both prognostic and predictive roles, although quantitative parameters (i.e., SUV_max_, SUV_mean_, [^18^F]FES/[^18^F]FDG ratio) were not uniform across included studies. On the one hand, the presence of functional ER activity, as visualized through [^18^F]FES PET/CT, is a significantly stronger predictor of therapy response than immunohistochemistry. Recent findings from an international multicenter randomized trial demonstrated that the SUV values from [^18^F]FES PET/CT in metastatic ER+HER2- breast cancer patients can identify distinct subgroups: those with endocrine-resistant disease who are likely to benefit from chemotherapy and those with endocrine-sensitive tumors for whom endocrine therapy alone is linked to exceptionally prolonged survival [28,29]. On the other hand, low or absent [^18^F]FES uptake is a reliable indicator of endocrine resistance [30,31].

When evaluating the role of [^18^F]FES PET in predicting the treatment response, the cut-off value of [^18^F]FES positivity is important and varied among several studies. Huang et al. found that the diagnostic performance and the rate of ER expression heterogeneity varied according to the chosen threshold. [^18^F]FES-positive lesions were more likely to respond compared with [^18^F]FES-negative ones. In particular, the threshold of SUV max of 1.5 to 2.0 presented 0.45 to 0.60 positive predictive value and 0.78 to 0.81 negative predictive value [32,33,34]. Similarly, [^18^F]FES PET was able to predict disease prognosis, demonstrating a longer median time to progression of disease in [^18^F]FES-positive lesions rather than in heterogeneous and negative lesions [21]. Nevertheless, the threshold of [^18^F]FES uptake remains a relevant parameter when evaluating single lesions. Using a threshold of SUV_mean_ greater than 1.5, the rate of treatment response was between 35% and 40%, and when SUV_mean_ was lower than 1.0, the rate of predicted endocrine resistance was 29% [15,31,35].

The development of resistance to endocrine therapy poses a major challenge in managing ER-positive breast cancer. By identifying patients whose tumors have lost functional ER activity, [^18^F]FES PET/CT can guide clinicians in transitioning to alternative treatment modalities, potentially improving patient outcomes. Few studies in our review evaluated the role of [^18^F]FES PET/CT in monitoring response to different types of therapy like tamoxifen, fulvestrant, vorinostat, and Z-endoxifen. These studies demonstrated a significant reduction in [^18^F]FES uptake following treatment, particularly with fulvestrant and Z-endoxifen, indicating decreased ER availability. Notably, fulvestrant led to a substantial reduction in ER expression and was associated with prolonged disease control in the majority of patients [18,21,36]. In contrast, vorinostat did not induce a systematic change in [^18^F]FES uptake, suggesting no significant effect on ER binding [37]. These findings support the complementary role of [^18^F]FES PET/CT alongside [^18^F]FDG PET/CT in assessing early response to endocrine therapy.

Subgroup analyses considering the type of lesions, the PET technique, the timing of the exam, the sample size, the histotype, the site of metastases, and the tumor size, along with the [^18^F]FES uptake, showed no significant difference in the diagnostic accuracy. When [^18^F]FES PET/CT is compared with [^18^F]FDG PET/CT, both methods are equally effective; however, the first has better performance for lobular tumors and bone metastases [14,20,38]. Lobular carcinoma is distinguished by its diffuse growth pattern, lower proliferation rate, and reduced tumor glycolysis compared to ductal carcinoma, and despite its relatively indolent nature, it is associated with a poor prognosis. This unique behavior poses specific challenges in diagnosis and treatment, raising concerns about the reliability of [^18^F]FDG PET for staging [39,40]. Previous studies, reporting a higher number of [^18^F]FDG-positive lesions, mainly included patients with ductal carcinomas and liver metastases. In the recently published joint EANM/SNMMI guidelines on the role of [^18^F]FDG PET/CT in no special type breast tumors, the authors emphasized the need for further evidence to establish guidelines or recommendations for the lobular subtype. They also recognized the potential appropriateness of using [^18^F]FES PET/CT for staging lobular and low-grade breast cancers, as well as other subtypes with low [^18^F]FDG uptake [41].

Notably, a change of treatment and management was reported in 20–48% of breast cancer patients undergoing [^18^F]FES PET/CT, suggesting that adding this exam into the routine diagnostic work-up may result in a modified therapeutic pathway [15,21,22]. Previous studies have already demonstrated that [^18^F]FDG PET/CT for initial clinical staging of breast tumors changed the treatment strategy, mainly in stage IIB and stage III patients [42,43,44]. [^18^F]FES PET/CT demonstrates exceptionally high sensitivity in detecting secondary lesions in organs such as bone, which is the most common site of metastasis in ER-positive breast cancer. In lobular tumors, it was able to detect additional breast and axillary lesions in 24% of patients when compared with standard imaging [45]. However, the main limitation of [^18^F]FES PET/CT is its high physiological uptake in the liver, which prevents it from detecting secondary lesions in this organ [20,24]. It remains a safe and non-invasive method for whole-body assessment of ER-positive lesions.

Lastly, the safety of [^18^F]FES was reliable, with 10% of patients feeling pain with drug injection and no other side effects reported [24]. The most common adverse reactions described by manufacturers occurred at a rate lower than 1% and were injection-site pain and dysgeusia.

The strengths of this umbrella review lie in its comprehensive synthesis of existing evidence, offering clinicians a valuable decision-making tool for the application of [^18^F]FES PET/CT in breast cancer management. Additionally, this review identified discrepancies and heterogeneity across various studies, reducing redundancy and highlighting research gaps for future investigation.

Although this review synthesizes current evidence, it is important to acknowledge potential limitations, including variability in study designs, imaging protocols, and patient populations across the included studies. Furthermore, being an umbrella review, it relies on the quality of the included reviews, with one of them demonstrating low methodological quality. Additionally, the analysis was restricted to evidence from pre-existing reviews, without incorporating primary data, so the lack of quantitative measures prevented the execution of further analyses. Certain outcomes, such as the pooled detection rate, statistical heterogeneity, and publication bias, were either limited or absent. The heterogeneity among the included reviews further complicates the generalization of results. Lastly, focusing on aggregate data at the review level may lead to the lack of patient-specific insights that could be valuable for individualized patient evaluation.

## 5. Conclusions

Our umbrella review highlights the significant role of [^18^F]FES PET/CT in breast cancer management, providing a non-invasive method to assess functional ER expression, explore its heterogeneity across primary and metastatic sites, and predict response to endocrine therapy. By offering a whole-body evaluation of ER status, [^18^F]FES PET/CT addresses key limitations of IHC, particularly in cases where biopsy is unfeasible or tumor heterogeneity complicates treatment decisions. This imaging modality has demonstrated utility in prognostic assessment and treatment monitoring, reinforcing its value as a complement to conventional diagnostic tools.

[^18^F]FES PET/CT offers a valuable approach for patients with ER-positive, well-differentiated, or lobular breast cancer, which often presents with low [^18^F]FDG uptake and may be challenging to evaluate with standard imaging techniques.

Future research should focus on standardizing imaging protocols, defining optimal uptake thresholds for clinical decision making, and integrating [^18^F]FES PET/CT into routine workflow. Expanding its application in prospective clinical trials will be essential to fully establish its impact on patient outcomes and solidify its role in precision medicine.

## Figures and Tables

**Table 1:** Quality assessment of the included systematic reviews.

N	AMSTAR-2 Criteria	Chae SY et al., 2019 [24]	Evangelista L et al., 2016 [18]	Huang YT et al., 2023 [21]	Kurland BF et al., 2020 [12]	Matushita CS et al., 2023 [22]	Mo JA 2021 [19]	Piccardo A et al., 2022 [20]	van Geel JJL et al., 2022 [23]
1	Research questions and inclusion criteria include components of PICO	yes	yes	yes	yes	yes	yes	yes	yes
2	Review methods established prior to the conduct of the review (protocol) and deviations justified	partial yes	partial yes	partial yes	partial yes	partial yes	partial yes	partial yes	partial yes
3	Selection of study design explained	yes	yes	yes	yes	yes	yes	yes	yes
4	Comprehensive literature search strategy	partial yes	partial yes	partial yes	partial yes	partial yes	partial yes	partial yes	partial yes
5	Study selection in duplicate	yes	yes	yes	not reported	yes	yes	yes	not reported
6	Data extraction in duplicate	yes	yes	not reported	yes	not reported	yes	yes	not reported
7	List of excluded studies and justification of the exclusions	partial yes	partial yes	partial yes	partial yes	partial yes	partial yes	partial yes	partial yes
8	Included studies described in adequate detail	yes	yes	yes	yes	yes	yes	yes	yes
9	Technique for assessing the risk of bias satisfactory	yes	yes	yes	yes	yes	yes	yes	yes
10	Sources of funding for the primary studies reported	no	no	no	no	no	no	no	no
11	Appropriate methods for meta-analysis	yes	yes	yes	yes	yes	yes	yes	yes
12	Potential impact of risk of bias results on meta-analysis assessed	yes	yes	yes	yes	yes	yes	yes	yes
13	Risk of bias results accounted for in discussion/conclusion	yes	yes	yes	yes	yes	no	yes	yes
14	Satisfactory discussion and explanation of observed heterogeneity, if any	yes	yes	yes	yes	yes	yes	yes	yes
15	Adequate investigation of publication bias	no	yes	no	no	no	no	yes	no
16	Conflict of interest of review authors and funding received for conducting the review reported	yes	yes	yes	yes	yes	yes	yes	yes
	Overall methodological quality	moderate	high	moderate	moderate	moderate	low	high	moderate

**Table 2:** Main characteristics of selected systematic reviews and/or meta-analyses on [^18^F]FES PET or PET/CT in patients with breast cancer.

Authors (Year of Publication)	Studies (Patients) Included in the Meta-Analysis	Pooled Sensitivity (95% CI)	Pooled Specificity (95% CI)	AUC	Pooled Detection Rate for Malignancy (95% CI)	Statistical Heterogeneity	Publication Bias
Evangelista et al. (2016) [18]	9 (238)	82% (74–88)	95% (86–99)	0.915	NR	NO	YES
Chae et al. (2019) [24]	5 (NR)	83% (72–91)	93% (74–99)	NR	NR	NO	NR
Kurland et al. (2020) [12]	11 (NR)	81% (73–87)	86% (68–94)	0.89	NR	YES	NR
Mo (2021) [19]	8 (284)	86% (80–91)	85% (76–92)	0.910	NR	NO	NR
Piccardo et al. (2022) [20]	7 (171)	94% (89–99)	NR	NR	NR	YES	NR
Van Geel et al. (2022) [23]	12 (556)	89% (85–92)	78% (69–84)	0.910	NR	NR	NR
Matushita et al. (2023) [22]	7 (NR)	82% (76–87)	94% (86–98)	0.889	NR	NO	NR
Huang et al. (2023) [21]	21 (NR)	NR	NR	NR	80% (75–85)	YES	NR

95% CI: 95% confidence interval; AUC: area under the receiver-operating characteristics curve; NR: not reported; CT computed tomography; FES fluoroestradiol; PET positron emission tomography.

## Data Availability

The data analyzed in this umbrella review can be found in the cited studies.

## Abbreviations

The following abbreviations are used in this manuscript:

AI	Aromatase inhibitor
AMSTAR-2	A Measurement Tool to Assess Systematic Reviews 2
BC	Breast cancer
CDK4/6	Cyclin-dependent kinase 4/6
CT	Computed tomography
ER	Estrogen receptor
FDG	Fluoro-2-deoxy-2-D-glucose
FDA	Food and Drug Administration
FES	Fluoroestradiol
HER2	Human epidermal growth factor receptor 2
IHC	Immunohistochemistry
MBq	Megabecquerel
OS	Overall survival
PET	Positron emission tomography
PFS	Progression-free survival
SUV	Standardized uptake value
SUVmax	Maximum standardized uptake value
SUVmean	Mean standardized uptake value
